# Voice Disorder in Cystic Fibrosis Patients

**DOI:** 10.1371/journal.pone.0096769

**Published:** 2014-05-05

**Authors:** Bruna Mendes Lourenço, Kauê Machado Costa, Manoel da Silva Filho

**Affiliations:** 1 Institute of Biological Sciences, Federal University of Pará, Belém, Pará, Brazil; 2 João de Barros Barreto University Hospital, Federal University of Pará, Belém, Pará, Brazil; University of Texas Health Science Center at San Antonio, Research Imaging Institute, United States of America

## Abstract

Cystic fibrosis is a common autosomal recessive disorder with drastic respiratory symptoms, including shortness of breath and chronic cough. While most of cystic fibrosis treatment is dedicated to mitigating the effects of respiratory dysfunction, the potential effects of this disease on vocal parameters have not been systematically studied. We hypothesized that cystic fibrosis patients, given their characteristic respiratory disorders, would also present dysphonic symptoms. Given that voice disorders can severely impair quality of life, the identification of a potential cystic fibrosis-related dysphonia could be of great value for the clinical evaluation and treatment of this disease. We tested our hypothesis by measuring vocal parameters, using both objective physical measures and the GRBAS subjective evaluation method, in male and female cystic fibrosis patients undergoing conventional treatment and compared them to age and sex matched controls. We found that cystic fibrosis patients had a significantly lower vocal intensity and harmonic to noise ratio, as well as increased levels of jitter and shimmer. In addition, cystic fibrosis patients also showed higher scores of roughness, breathiness and asthenia, as well as a significantly altered general grade of dysphonia. When we segregated the results according to sex, we observed that, as a group, only female cystic fibrosis patients had significantly lower values of harmonic to noise ratio and an abnormal general grade of dysphonia in relation to matched controls, suggesting that cystic fibrosis exerts a more pronounced effect on vocal parameters of women in relation to men. Overall, the dysphonic characteristics of CF patients can be explained by dysfunctions in vocal fold movement and partial upper airway obstruction, potentially caused by the accumulation of mucus and chronic cough characteristic of CF symptomatology. Our results show that CF patients exhibit significant dysphonia and suggest they may potentially benefit from voice therapy as a parallel treatment strategy.

## Introduction

Voice disorders, or dysphonias, can be caused by neurological, structural or functional problems affecting breathing, phonation, articulation, resonance and prosody or the production of sounds at the level of the larynx [1]. A variety of respiratory tract disorders, including chronic cough and laryngeal tension, can lead to reduced voice quality [2]. Dysphonias can cause severe drawbacks in communication and significantly impair quality of life [3], [4]. Given the importance of vocal communication to human interaction, subjects with abnormal voice patterns are at high risk for anxiety disorders and depression [5]. Patients with voice disorders score significantly lower on all health status subscales, as measured by the SF-36 form, a highly validated questionnaire assessing quality of life [4].

Cystic fibrosis (CF) is a common autosomal recessive disorder, with a prevalence of approximately 1∶2500 live births in people of European descent [6]. This disease is caused by mutations in the cystic fibrosis transmembrane regulator gene, which encodes an epithelial chloride channel. Dysfunctions in this ion channel lead to abnormal transport of chloride and sodium across epithelial membranes and result in the production of thick, viscous secretions in various organs, including the respiratory tract [6], [7]. Respiratory dysfunctions, such as shortness of breath and chronic cough, are a hallmark of CF and a major cause of morbidity in CF patients. Therefore, in current medical practice a large part of CF treatment is dedicated to mitigating the effects of respiratory dysfunction [7], [8]. However, the potential effects of CF respiratory complications on the vocal capacity of CF patients have not been studied in detail.

We hypothesized that CF patients, given their characteristic respiratory disorders, would also present intrinsic voice alterations. If this is true, CF patients could potentially benefit from voice therapy as a way to improve their quality of life. We tested our hypothesis by measuring vocal parameters, using both objective and subjective clinical evaluation methods, in male and female CF patients undergoing conventional treatment and compared them to age and sex matched controls.

## Methods

### Subject selection and Ethics Statement

Subjects aged between 10 and 30 years were divided into four groups: a male control group (n = 25), a female control group (n = 16), male patients with CF (n = 15) and female patients with CF (n = 8). For some analyses, CF patients (n = 23) and control subjects (n = 41) were pooled independently of their sex. CF patients were diagnosed based on the standard sweat test, family history and the presence of chronic pulmonary disease and pancreatic insufficiency. Previous laryngoscopic examination (conducted prophylactically as part of the patients' clinical monitoring) confirmed that the selected CF patients had no drastic vocal cord dysfunctions or laryngeal diseases, such as vocal cord polyps, laryngeal carcinoma or laryngeal paralysis. CF patients and control subjects had never received voice therapy.

Patients with CF were receiving, individually, regular treatment accompanied by physician, dietician, physiotherapist and psychologist. At the time of the study, none of the CF patients had signs of infection and were not being medicated with inhalatory antibiotics or hypertonic saline. Further information on the clinical status of the subjects can be found in Tables S1–S4, including body mass index (BMI) and FEV1 (a common measure of lung function defined as the volume of air that is exhaled in the first second of forced expiration and normalized by the mean values for the population). The low number of CF patients of each sex is a limitation of this study and was due to the limited pool of willing CF patients which fitted the necessary criteria. This study was approved by the research ethics committee of the João de Barros Barreto University Hospital (#112/09) and all subjects or their legal caretakers signed an informed consent form.

### Vocal recordings

Subjects were asked to produce a sustained/a/vowel phonation in their usual intensity for a maximum voicing time. Voice recordings were conducted using a hand-held microphone (PG42-LC, Shure) always positioned six centimeters from the subject's mouth and at a 45° angle to ensure comparable intensity measurements. The initial second of the recording was always excluded from the analysis and parameters were measured over the three second time window that followed this period. Signals were recorded with a sampling rate of 44.1 kHz and analyzed with the Praat 5.1.36 software.

### Objective vocal analysis

We measured the following quantitative vocal parameters: fundamental frequency (F_0_), intensity, jitter (index of F_0_ variability), shimmer (index of intensity variability) and the harmonics to noise ratio (HNR; index of glottal turbulence noise and hoarseness). These parameters have been extensively validated as quantitative, objective measures of vocal quality and are routinely used by voice care professionals as diagnostic tools [9]. Low values of vocal intensity and HNR, high values of jitter and shimmer and altered F_0_ are all potential signs of dysphonia.

### Subjective vocal analysis

In addition to these objective acoustic variables, we also conducted a perceptual assessment of voice quality in CF and control subjects using the GRBAS (Grade, Roughness, Breathiness, Asthenia, Strain) scale [10]. This vocal assessment method evaluates a patient's general grade of dysphonia (represented by the letter G) through four subjective parameters: roughness (R), breathiness (B), asthenia (A), and strain (S). These variables are determined by a voice care professional, who quantifies his perception of the patients voice as zero (normal), one (mild alteration), two (moderate alteration) or three (severe alteration) on each of the abovementioned factors. The reliability of this method has been extensively validated [10]–[13]. In order to avoid any confirmation bias in our analyses, we asked an independent practicing clinical voice therapist, with no ties to the research project, to perform the GRBAS evaluation of all our recordings. The evaluator received the recordings in a pseudo-randomized order and was completely blind to the structure of the experimental protocol, the project's objective and the identification and diagnosis of each subject. The evaluator had 9 years of professional experience in voice therapy and GRBAS analysis methods and was instructed to analyze our recordings exactly as it would be performed in clinical practice.

### Data analysis and statistics

Differences in objective and subjective vocal parameters were evaluated with the Mann-Whitney test. Data distribution was non-Gaussian for many variables, as defined by the Shapiro-Wilk normality test (Table S5), and therefore data are represented as median values or median and interquartile range (IQR). Statistical significance level was initially set at *P*<0.05; to account for multiple comparisons within the objective and subjective categories of vocal parameters, the Bonferroni correction method was applied. This procedure adjusted the significance level to *P*<0.01 for all analyses.

## Results

### Representative recordings

**Figure 1:**
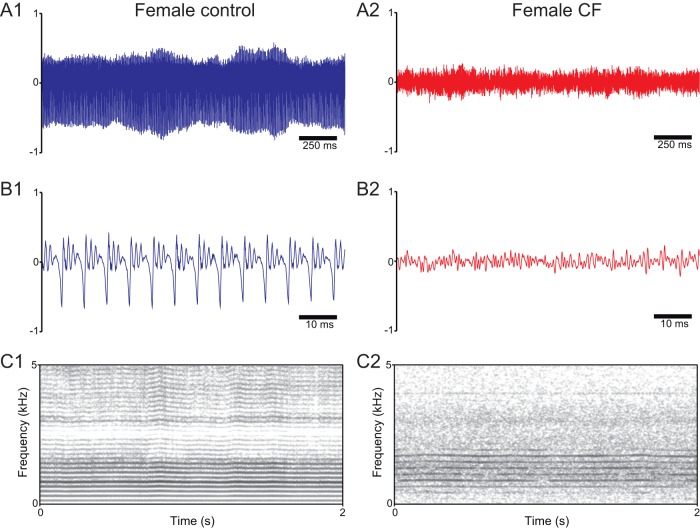
Representative voice recordings and spectrograms of female patients with CF (red) and healthy controls (blue). A: Sound recordings of/a/vowel phonations from a female control subject (A1) and a female patient with CF (A2); note the markedly reduced amplitude range of the signal from the CF patient in relation to the control. B: Amplified views of the recordings presented in A1 and A2; note the irregularity of the voice signal from the CF patient in relation to the control. C: Spectrogram (frequency domain) representations of the recordings presented in A1 and A2; note the higher level of background noise and low formant segregation in the CF patient in relation to the healthy control.

**Figure 2:**
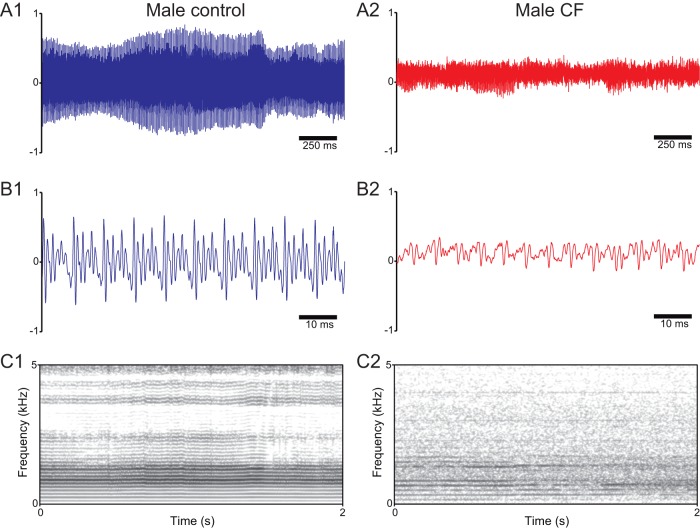
Representative voice recordings and spectrograms of male patients with CF (red) and healthy controls (blue). A: Sound recordings of/a/vowel phonations from a male control subject (A1) and a male patient with CF (A2); note the markedly reduced amplitude range of the signal from the CF patient in relation to the control. B: Amplified views of the recordings presented in A1 and A2; note the irregularity of the voice signal from the CF patient in relation to the control. C: Spectrogram (frequency domain) representations of the recordings presented in A1 and A2; note the higher level of background noise and low formant segregation in the CF patient in relation to the healthy control.

Voice signal recordings and spectrograms of representative individuals from all studied groups are shown in Figures 1 and 2. In the control group, recordings show clear features of a euphonic voice [14], such as high periodicity and wide amplitude ranges (Figures 1-B1 and 2-B1). In contrast, signals from subjects in the CF group showed remarkable aperiodicity and reduced amplitude (Figures 1-B2 and 2-B2). The spectrograms of CF patients also showed high noise levels and the loss of high frequency harmonics in relation to the controls (Figures 1-C2 and 2-C2).

### CF-related changes in vocal parameters

**Figure 3:**
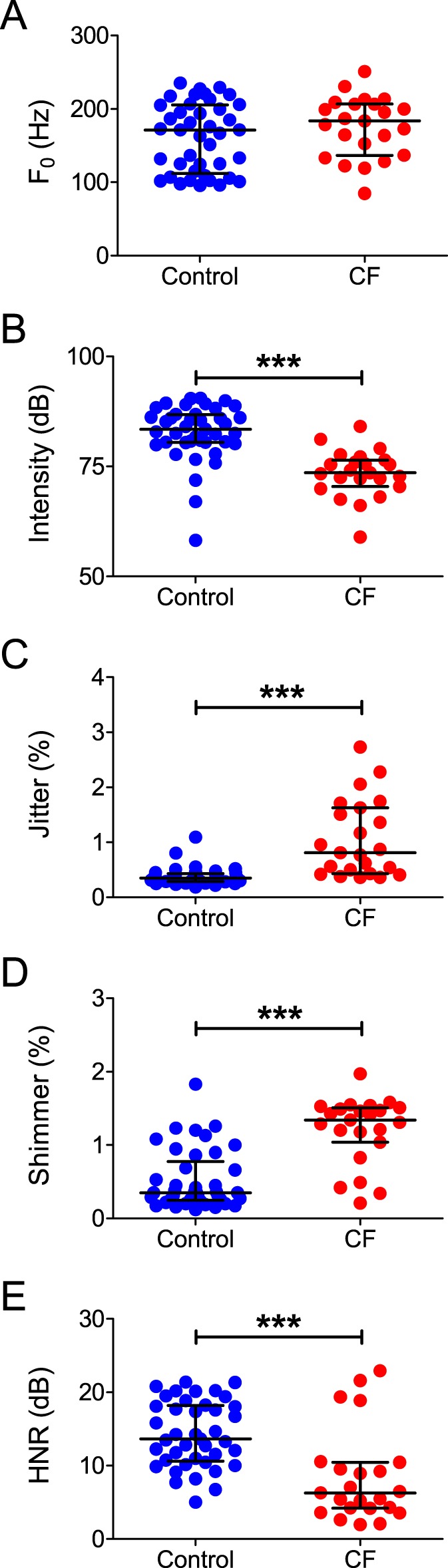
Objective measurements of vocal parameters of control subjects and CF patients. Horizontal lines and error bars represent median±IQR for all variables. Subjects were pooled independently of their sex. **A:** Values of F_0_ for each group. **B:** Values of intensity for each group; note the significant reduction in intensity in the CF group. **C:** Values of jitter for each group; note the significant increase in jitter in the CF group. **D:** Values of shimmer for each group; note the significant increase in shimmer in the CF group. **E:** Values of HNR for each group; note the significant reduction in this variable in the CF group. ***  = *P*<0.0001.

Analysis of objective voice parameters revealed that CF patients presented a significant decrease in intensity (Figure 3-B; *P*<0.0001), an increase in jitter (Figure 3-C: *P*<0.0001) and shimmer (Figure 3-D; *P*<0.0001) and a much lower HNR (Figure 3-E; *P*<0.0001) in relation to controls. No differences were observed in F_0_ (Figure 3-A; *P*>0.01) between the two groups.

**Figure 4:**
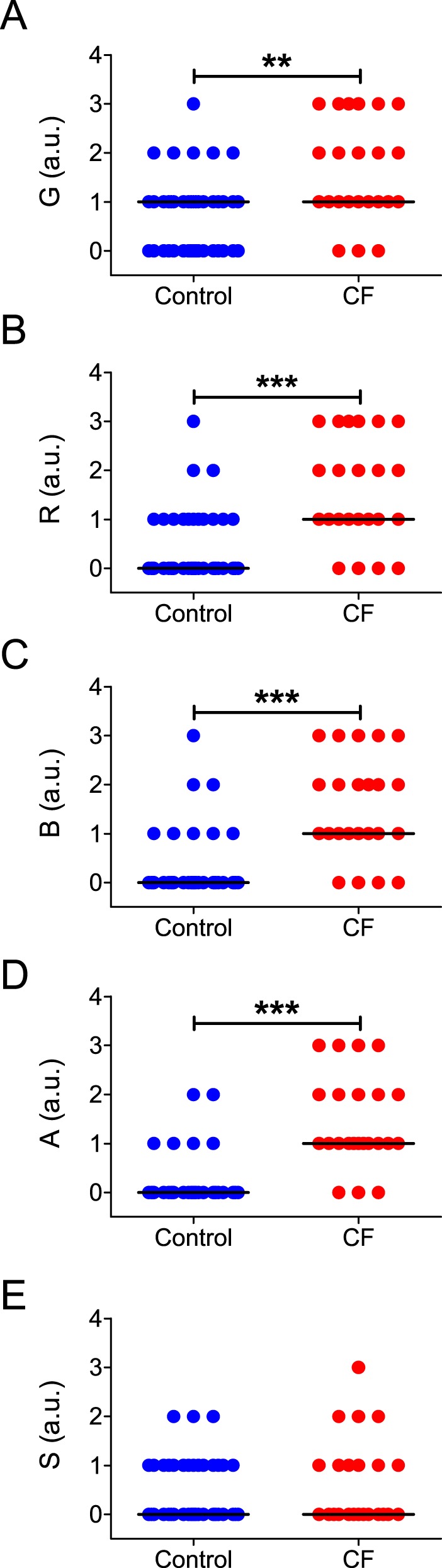
Subjective measurements of vocal parameters of male and female control subjects and CF patients. Horizontal lines and error bars represent the median value of each group. Subjects were pooled independently of their sex. **A:** Values of G for each group; note the significant increase in G in the CF group. **B:** Values of R for each group; note the significant increase in R in the CF group. **C:** Values of B for each group; note the significant increase in B in the CF group. **D:** Values of A for each group; note the significant increase in A in the CF group. **E:** Values of S for each group. **  = *P*<0.001; ***  = *P*<0.0001.

The perceptual assessment of voice quality on the GRBAS scale also revealed clear signs of dysphonia in CF patients. In relation to the control group, patients with CF presented an overall increase in R (Figure 4-B; *P*<0.0001), B (Figure 4-C; *P*<0.0001) and A (Figure 4-D; *P*<0.0001). In addition, CF patients showed an increase in G in relation to controls (Figure 4-A; *P* = 0.0012). Interestingly, no difference was observed in the S score between CF patients and healthy controls (Figure 4-E; *P*>0.01). All median values and IQRs for the studied variables and statistical comparisons between groups are summarized in Table S6.

It is important to note that it is highly unlikely that the CF-related changes in vocal parameters are related to the age or lung function of the subjects, as none of the objective variables that were affected in CF patients had any significant correlation with age or FEV1 (Figures S1 and S2; r2<0.2 and *P*<0.01 for correlations of age or FEV1 with intensity, jitter, shimmer and HNR).

### Influence of sex in CF-related dysphonia

**Figure 5:**
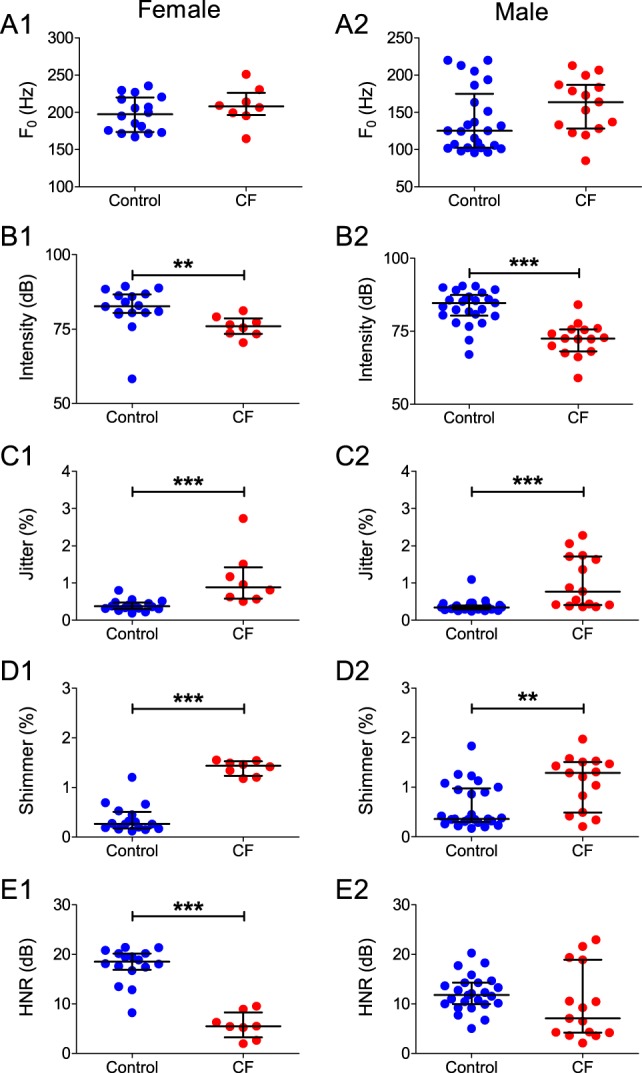
Objective measurements of vocal parameters of male and female control subjects and CF patients. Horizontal lines and error bars represent median±IQR for all variables. **A:** Values of F_0_ for female (A1) and male (A2) subject groups. **B:** Values of intensity for female (A1) and male (A2) subject groups. **C:** Values of jitter for female (A1) and male (A2) subject groups. **D:** Values of shimmer for female (A1) and male (A2) subject groups. **E:** Values of HNR for female (A1) and male (A2) subject groups; note that only female CF patients have a lower HNR in relation to the matched controls. **  = *P*<0.001; ***  = *P*<0.0001.

We also analyzed our data segregating the control and CF groups according to sex, in order to reveal any possible influence of sex in CF-related dysphonia. These analyses revealed that both male and female CF patients, in relation to their matched controls, presented a significant decrease in intensity (Figure 5-B1 and 5-B2; *P*<0.0001 for both males and females) and an increase in jitter (Figure 5-C1 and 5-C2; for males: *P* = 0.001 for females and *P*<0.0001 for males) and shimmer (Figure 5-D1 and 5-D2; *P* = 0.0001 for females and *P* = 0.0038 for males). Interestingly, while female CF patients showed drastically lower values of HNR (Figure 5-E1; *P* = 0.0002) in relation to the control group, this feature was not observed in male CF patients (Figure 5-E2; *P*>0.01) compared to their respective controls. No differences between CF patients and control subjects were observed in F_0_ for both sexes (Figure 5-A1 and 5-A2; *P*>0.01 for both comparisons).

**Figure 6:**
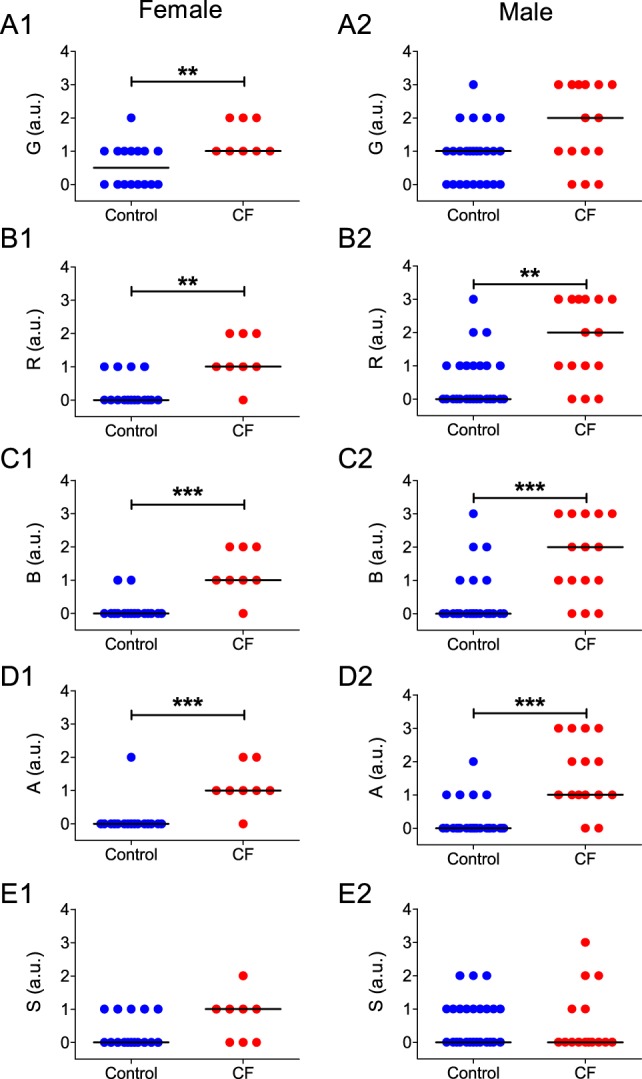
Subjective measurements of vocal parameters of male and female control subjects and CF patients. Horizontal lines and error bars represent the median value of each group. **A:** Values of G for female (A1) and male (A2) subject groups; note that only female patients have a significant change in G in relation to the matched controls. **B:** Values of R for female (A1) and male (A2) subject groups. **C:** Values of B for female (A1) and male (A2) subject groups. **D:** Values of A for female (A1) and male (A2) subject groups. **E:** Values of S for female (A1) and male (A2) subject groups. **  = *P*<0.001; ***  = *P*<0.0001.

In relation to the subjective vocal parameters analyzed on the GRBAS scale, both male and female patients with CF presented an overall increase in R (Figure 6-B1 and 6-B2; *P* = 0.0019 for females and *P* = 0.0025 for males), B (Figure 6-C1 and 6-C2; *P* = 0.0003 for females and *P* = 0.0004 for males) and A (Figure 6-D1 and 6-D2; *P* = 0.0003 for females and *P*<0.0001 for males) compared to matched controls. However, only female CF patients showed an increase in G in relation to controls (Figure 6-A1; *P* = 0.0081), which was not observed in male subjects (Figure 6-A2; *P* = 0.0293). No difference was observed in the S score between CF patients and healthy controls of both sexes (Figure 6-E1 and 6-E2; *P*>0.01 for both comparisons). All median values and IQRs for the studied variables and statistical comparisons between CF patients and control subjects segregated by sex are summarized in Table S7.

## Discussion

Our results show that subjects with CF present significantly altered vocal parameters, both objectively and subjectively. To our knowledge, this is the first systematic investigation of voice dysfunction in CF. Our findings suggest that dysphonia is a relevant manifestation of CF pathology.

Objective vocal parameters affected by CF were intensity, jitter, shimmer and HNR. Changes in vocal intensity can be observed in cases of vocal microtrauma, neurological dysfunction and obstructive respiratory diseases [15]. Vocal intensity depends fundamentally of subglottal air pressure, upper airway airflow and glottal resistance [16]. The observed decrease in intensity in CF patients could thus be explained by the inefficient lung function and partial upper airway obstruction characteristic of CF physiopathology [6], [8]. Chronic cough, a major symptom of CF, could also contribute to this effect [2], [6], [7], [15].

Changes in jitter and shimmer are typically related to irregularities in vocal fold vibration, and are also usually related to vocal harshness and the presence of noise in the vocal signal [9], [17], [18]. The differences in these variables observed in our dataset can be due to the presence of thick mucous secretions in the vocal folds of CF patients, which can potentially interfere in the oscillatory motion of the vocal folds [19], [20]. In addition, chronic cough is also known to cause dysfunction of vocal fold movement [2].

Decreases in HNR are caused by increases in the variation of vocal amplitude and frequency and in upper airway turbulence. This produces distortions in the vocal signal, emphasizing the sub-harmonic components and vocal breaks and, consequently, reducing the relative contribution of harmonic components to the overall vocal signal [21]. More importantly, it has been reported that high vocal noise (low HNR) is the most characteristic sign of dysphonia [13]. CF patients showed drastically low HNR values, which can be considered pathologically hoarse and highly detrimental to a normal voice production [15], [22]. This decrease in HNR could have the same physiological underpinning as the changes in jitter and shimmer, namely the accumulation of mucus and chronic cough.

The changes in objective vocal parameters in CF patients were confirmed in the subjective evaluation of vocal quality using the GRBAS scale. CF patients showed significant differences in most perceptual vocal parameters, with the exception of S [23]. Previous studies have shown that increases in R and B are strongly related with increases in shimmer [23], which is in agreement with our finding that all three variables are higher in CF patients in relation to the controls. Increases in A are caused by decreases in airflow rate in the upper airways, a common feature of many respiratory diseases [24]. In these cases, subglottal pressure does not rise to the appropriate levels during voice production, causing asthenia and hoarseness. In our dataset of CF patients, this feature can be related to the observed decrease in intensity.

Differences in G represent the general degree of vocal disorder, i.e. a subjective summation of all other perceptual parameters [11], [12]. This makes G the most important index within the GRBAS scale [10]. The significantly higher values of G in CF patients indicated that the individual changes in R, B and A combine to produce a noticeable condition of dysphonia.

A notable finding of our study is that female CF patients appear to have more vocal alterations in relation to the controls than male CF patients. When data from each sex was analyzed separately, only female CF patients presented significant lower values of HNR, and higher values of G compared to matched controls. This difference between sexes may be due to the intrinsic differences in the structure of the vocal apparatus observed between men and women. On average, men have thicker vocal folds, larger glottal area and airflow, and lower laryngeal airway resistance than women [25]–[28]. Our data show that CF vocal pathology is probably due to disorders in vocal fold oscillation and obstruction of the upper airways, and these dysfunctions can be exacerbated when associated to thinner vocal folds and higher airway resistance, respectively. Taken together, these gender-related structural differences are likely to be a major factor in making female patients more susceptible to CF-related dysphonia.

In addition, several other studies have reported that healthy males have voices with significantly lower HNR than healthy females across different languages and age ranges [29]–[32]. This is also the case in our dataset, as recordings from male control subjects had significantly lower HNRs than female control subjects (Figure 5-E1 and 5-E2; *P* = 0.0001 between female and male controls). This intrinsic difference might be explained both by the already mentioned gender related differences in the structure of the vocal apparatus and by social influences, as some authors speculate that women have better vocal training than men, as they use their voices more frequently [29]. Because the perception of what is a normal or appropriate voice depends on what is expected from a person's gender, age and social group, deviations in vocal parameters must be compared with matched controls. Indeed, because healthy women might be expected to have higher HNRs than men, it is fair to predict that the significantly larger decrease in HNR observed in female CF patients might have a highly deleterious impact in their quality of life.

Our data suggests that, as a group, only female patients have their voices affected by the pathophysiology of CF to a degree in which the general perception of their voice, and presumably its communication function, is significantly affected. Nevertheless, it is important to point out that 6 out of 15 subjects in the male CF patient group also presented very low values of HNR (under 5dB), even though the median values of the group were not significantly different from the control. In addition, 6 male CF subjects also presented levels of G above 2, while no such values were observed in the male control group. This would mean that, even though female CF patients might be more susceptible to the deleterious effects of CF on vocal capacity (and thus present more dysphonic symptoms than males), some individual male CF patients might also develop significant changes in HNR and G.

Our results open the possibility that CF patients may benefit from voice therapy as a clinical intervention strategy to reduce dysphonia and increase life quality. Voice therapy consists of a series of techniques applied to the mitigation of voice disorders, which include enhancing the patient's awareness of his or her dysphonia, application of vocal health guidelines, hearing training, muscle relaxation and psychodynamic vocal training [33], [34]. This form of therapy has been shown to be an effective treatment in reducing dysphonic symptoms in several diseases, including respiratory disorders, and is capable of at least partially restoring quality of life to affected patients [35]–[37].

The dysphonic characteristics observed in CF patients can be potentially attributed to dysfunctions in vocal fold movement and partial upper airway obstruction, both of which can be caused by the accumulation of mucus and chronic cough, which are standard symptoms of CF respiratory pathology. While there is no available data on which therapy would be adequate for CF patients, therapy for vocal disorders with hyperfunctional origins have been shown to be effective in patients with chronic cough [2]. Given that chronic cough is a major manifestation of CF pathology, this strategy could potentially be applied to CF patients. This type of intervention is usually based on voice education and vocal hygiene as strategies for cough suppression and also applies exercises that improve dysfunctional vocal fold vibration [2], which according to our data are probably the major features of CF-related dysphonia. Of course, further studies must be conducted to confirm the efficacy of this form of treatment, as well as any other form of voice therapy, for patients with CF.

## Conclusion

We showed that patients with CF exhibit symptoms of dysphonia, with changes in both objective and subjective vocal parameters. This may be a relevant feature of CF pathology and morbidity. Voice alterations were more pronounced in female patients, which exhibited a marked reduction in both HNR and overall subjective voice quality. Our results suggest that CF patients might potentially benefit from voice therapy.

## Supporting Information

Figure S1 **Linear correlation analyses between age and objective vocal parameters for the control and CF groups.** *P* values indicate if the slope of the linear regression line is significantly different from zero. **A:** Linear correlation between age and F_0_ for each group; note that there is a relatively high inverse correlation (r^2^ around 0.2) between these two variables in both the control and CF groups. **B:** Linear correlation between age and intensity for each group. **C:** Linear correlation between age and jitter for each group. **D:** Linear correlation between age and shimmer for each group. **E:** Linear correlation between age and HNR for each group. **  = *P*<0.001.
(EPS)

Figure S2 **Linear correlation analyses between FEV1 and objective vocal parameters in the CF group.** FEV1 is a common measure of lung function, defined as the volume of air that is exhaled in the first second of forced expiration and normalized to the mean values for the population. *P* values indicate if the slope of the linear regression line is significantly different from zero. Note that only very small correlations were found between FEV1 and each objective vocal parameter. **A:** Linear correlation between FEV1 and F_0_ for each group. **B:** Linear correlation between FEV1 and intensity. **C:** Linear correlation between FEV1 and jitter. **D:** Linear correlation between FEV1 and shimmer. **E:** Linear correlation between FEV1 and HNR.
(EPS)

Table S1 **Age and body mass index (BMI) for all subjects of the female control group.**
(DOCX)

Table S2 **Age and body mass index (BMI) for all subjects of the male control group.**
(DOCX)

Table S3 **Age, body mass index (BMI), FEV1 and medical status of the female cystic fibrosis group.**
(DOCX)

Table S4 **Age, body mass index (BMI), FEV1 and medical status of the male cystic fibrosis group.**
(DOCX)

Table S5 **Summary of mean ± standard error values and normality test results for all studied groups.**
(DOCX)

Table S6 **Summary of median and IQR values for pooled data of CF and control subjects, with statistical comparison including values for the Mann-Whitney U (U), Z-score (Z), effect size (r) and P.**
(DOCX)

Table S7 **Summary of median and IQR values for data of CF and control subjects segregated according to sex, with statistical comparison including values for the Mann-Whitney U (U), Z-score (Z), effect size (r) and P.**
(DOCX)
