# Supplementary material for: Voice Disorder in Cystic Fibrosis Patients
Source: PLoS One. 2014 May 5;9(5):e96769. doi: 10.1371/journal.pone.0096769 (PMC4010511; doi:10.1371/journal.pone.0096769)
Supplement: Table S1 — Age and body mass index (BMI) for all subjects of the female control group. (DOCX) [file pone.0096769.s003.docx]

**Table S1. Age and body mass index (BMI) for all subjects of the female control group.**

| Subject | Age (years) | BMI |
| --- | --- | --- |
| CTRLF1 | 26 | 22.3 |
| CTRLF2 | 10 | 22.83 |
| CTRLF3 | 30 | 24.2 |
| CTRLF4 | 11 | 20 |
| CTRLF5 | 23 | 18.2 |
| CTRLF6 | 13 | 20.5 |
| CTRLF7 | 12 | 17.9 |
| CTRLF8 | 12 | 22.2 |
| CTRLF9 | 21 | 20.7 |
| CTRLF10 | 29 | 21.8 |
| CTRLF11 | 11 | 15.1 |
| CTRLF12 | 28 | 21.6 |
| CTRLF13 | 14 | 21.5 |
| CTRLF14 | 26 | 23.1 |
| CTRLF15 | 13 | 17.3 |
| CTRLF16 | 30 | 24.8 |
