# Supplementary material for: Voice Disorder in Cystic Fibrosis Patients
Source: PLoS One. 2014 May 5;9(5):e96769. doi: 10.1371/journal.pone.0096769 (PMC4010511; doi:10.1371/journal.pone.0096769)
Supplement: Table S2 — Age and body mass index (BMI) for all subjects of the male control group. (DOCX) [file pone.0096769.s004.docx]

**Table S2. Age and body mass index (BMI) for all subjects of the male control group.**

| Subject | Age (years) | BMI |
| --- | --- | --- |
| CTRLM1 | 24 | 35.9 |
| CTRLM2 | 21 | 20.2 |
| CTRLM3 | 13 | 18.6 |
| CTRLM4 | 11 | 19.1 |
| CTRLM5 | 22 | 29.6 |
| CTRLM6 | 22 | 26.5 |
| CTRLM7 | 13 | 19.8 |
| CTRLM8 | 12 | 15.3 |
| CTRLM9 | 29 | 29.3 |
| CTRLM10 | 17 | 19.9 |
| CTRLM11 | 12 | 19.5 |
| CTRLM12 | 12 | 29.9 |
| CTRLM13 | 12 | 16.8 |
| CTRLM14 | 26 | 26.5 |
| CTRLM15 | 30 | 28.4 |
| CTRLM16 | 29 | 20.7 |
| CTRLM17 | 12 | 21.1 |
| CTRLM18 | 26 | 19.4 |
| CTRLM19 | 21 | 26.3 |
| CTRLM20 | 14 | 18.5 |
| CTRLM21 | 29 | 24.2 |
| CTRLM22 | 14 | 20.9 |
| CTRLM23 | 25 | 28.3 |
| CTRLM24 | 16 | 21.3 |
| CTRLM25 | 30 | 27.1 |
