# Supplementary material for: Voice Disorder in Cystic Fibrosis Patients
Source: PLoS One. 2014 May 5;9(5):e96769. doi: 10.1371/journal.pone.0096769 (PMC4010511; doi:10.1371/journal.pone.0096769)
Supplement: Table S3 — Age, body mass index (BMI), FEV1 and medical status of the female cystic fibrosis group. (DOCX) [file pone.0096769.s005.docx]

**Table S3. Age, body mass index (BMI), FEV1 and medical status of the female cystic fibrosis group.**

| Subject | Age (years) | BMI | FEV1(%) | Airway abnormalities | Medication at time of recording |
| --- | --- | --- | --- | --- | --- |
| CFF1 | 22 | 18.4 | 83.8 | None | Vitamin supplements |
| CFF2 | 11 | 26.4 | 37.5 | None | Pulmozyme |
| CFF3 | 30 | 19.7 | 42.11 | Deviated septum (left) | Pulmozyme  Digestive enzymes  Sulfamethoxazole  Formoterol  Azithromycin |
| CFF4 | 16 | 22.5 | 80.6 | None | Ultrase  Vitamin supplements  Pulmozyme |
| CFF5 | 11 | 17.5 | 81.7 | None | Ultrase  Vitamin supplements  Pulmozyme  Alinia |
| CFF6 | 13 | 21.8 | 68.7 | None | Ultrase  Vitamin supplements |
| CFF7 | 14 | 19.4 | 83.5 | None | Vitamin supplements |
| CFF8 | 10 | 21.6 | 70.7 | None | Ultrase  Pulmozyme  Prednisone |
