# Supplementary material for: Voice Disorder in Cystic Fibrosis Patients
Source: PLoS One. 2014 May 5;9(5):e96769. doi: 10.1371/journal.pone.0096769 (PMC4010511; doi:10.1371/journal.pone.0096769)
Supplement: Table S4 — Age, body mass index (BMI), FEV1 and medical status of the male cystic fibrosis group. (DOCX) [file pone.0096769.s006.docx]

**Table S3. Age, body mass index (BMI), FEV1 and medical status of the male cystic fibrosis group.**

| Subject | Age (years) | BMI | FEV1(%) | Airway abnormalities | Medication at time of recording |
| --- | --- | --- | --- | --- | --- |
| CFM1 | 13 | 15.9 | 45.3 | Chronic sinusitis | Vitamin supplements  Pulmozyme  Ultrase |
| CFM2 | 29 | 21.1 | 71.6 | None | Ultrase  Pulmozyme |
| CFM3 | 11 | 15.7 | 58.9 | None | Vitamin supplements |
| CFM4 | 16 | 18.7 | 63.5 | None | Vitamin supplements  Pulmozyme  Ultrase |
| CFM5 | 14 | 18.5 | 77.9 | None | Vitamin supplements |
| CFM6 | 19 | 23.8 | 91 | Cyst in right nostril | Vitamin supplements  Ultrase |
| CFM7 | 11 | 16.2 | 84.8 | None | Vitamin supplements  Digestive enzymes |
| CFM8 | 17 | 20.1 | 85.7 | None | Ultrase  Pulmozyme |
| CFM9 | 19 | 25.4 | 83.7 | None | Vitamin supplements  Pulmozyme  Ultrase |
| CFM10 | 13 | 17.4 | 87.4 | None | Digestive enzymes |
| CFM11 | 22 | 19.6 | 22 | None | Pulmozyme |
| CFM12 | 17 | 24.4 | 98.5 | Deviated septum (left) | Vitamin supplements  Pulmozyme  Ultrase  Budesonide |
| CFM13 | 12 | 15.8 | 81.4 | Deviated septum (left) | Vitamin supplements |
| CFM14 | 16 | 25 | 115.6 | None | Vitamin supplements |
| CFM15 | 13 | 17 | 63.2 | None | Vitamin supplements  Pulmozyme  Ultrase  Digestive enzymes |
