# Supplementary material for: Voice Disorder in Cystic Fibrosis Patients
Source: PLoS One. 2014 May 5;9(5):e96769. doi: 10.1371/journal.pone.0096769 (PMC4010511; doi:10.1371/journal.pone.0096769)
Supplement: Table S5 — Summary of mean ± standard error values and normality test results for all studied groups. (DOCX) [file pone.0096769.s007.docx]

**Table S5. Summary of mean ± standard error values and normality test results for all studied groups.**

| Variable | Pooled Control | *P* | Pooled CF | *P* | Female control | *P* | Female CF | *P* | Male control | *P* | Male CF | *P* |
| --- | --- | --- | --- | --- | --- | --- | --- | --- | --- | --- | --- | --- |
| F_0_ | 161.6±7.26 | 0.0034* | 176.2±8.54 | 0.6735 | 197.7±5.86 | 0.9187 | 208.8±8.99 | 0.8853 | 138.5±8.58 | 0.0009* | 158.8±9.60 | 0.7186 |
| Intensity | 82.71±1.00 | 0.0001* | 73.47 ± 1.06 | 0.5693 | 81.95±1.85 | 0.0008* | 75.83±1.21 | 0.9948 | 83.19±1.20 | 0. 0608 | 72.21±1.49 | 0.7137 |
| Jitter | 0.3810±0.0247 | < 0.0001* | 1.051±0.1453 | 0.0082* | 0.3913±0.0371 | 0.1194 | 1.108±0.2610 | 0.034 | 0.3744±0.0333 | < 0.0001* | 1.021±0.1803 | 0.0125* |
| Shimmer | 0.517±0.0628 | < 0.0001* | 1.209±0.0947 | 0.0058* | 0.371±0.0701 | 0.0017* | 1.398±0.0510 | 0.2169 | 0.6104±0.0885 | 0.0006* | 1.109±0.1373 | 0.2053 |
| HNR | 14.31±0.7001 | 0.1342 | 8.44±1.316 | 0.0008* | 17.75±0.8865 | 0.0131* | 5.688±0.940 | 0.4937 | 12.11±0.712 | 0.9275 | 9.904±1.870 | 0.0146* |
| G | 0.7561±0.9983 | < 0.0001* | 1.6090±0.2154 | 0.0048* | 0.5625±0.1573 | 0.0006* | 1.375±0.1830 | 0.0005* | 0.8800±0.1665 | 0.0008* | 1.733±0.3157 | 0.0074* |
| R | 0.4390±0.1107 | < 0.0001* | 1.5650±0.2252 | 0.0056* | 0.2500±0.1118 | < 0.0001* | 1.250±0.2500 | 0.0555 | 0.5600±0.1641 | < 0.0001* | 1.733±0.3157 | 0.0074* |
| B | 0.2927±0.1062 | < 0.0001* | 1.522±0.2166 | 0.0097* | 0.1250±0.0854 | < 0.0001* | 1.250±0.2500 | 0.0555 | 0.4000±0.1633 | < 0.0001* | 1.667±0.3034 | 0.0197* |
| A | 0.1951±0.0798 | < 0.0001* | 1.435±0.1971 | 0.0040* | 0.1250±0.1250 | < 0.0001* | 1.125±0.2266 | 0.0370 | 0.2400±0.1046 | < 0.0001* | 1.6000±0.2726 | 0.0309* |
| S | 0.4878±0.0995 | < 0.0001* | 0.6522±0.1845 | < 0.0001* | 0.3125±0.1197 | < 0.0001* | 0.7500±0.2500 | 0.0555 | 0.6000±0.1414 | < 0.0001* | 0.6000±0.2545 | 0.0001* |

*Statistically non-Gaussian according to the P<0.01 significance level and tested with the Shapiro-Wilk normality test.
