# Supplementary material for: Voice Disorder in Cystic Fibrosis Patients
Source: PLoS One. 2014 May 5;9(5):e96769. doi: 10.1371/journal.pone.0096769 (PMC4010511; doi:10.1371/journal.pone.0096769)
Supplement: Table S6 — Summary of median and IQR values for pooled data of CF and control subjects, with statistical comparison including values for the Mann-Whitney U (U), Z-score (Z), effect size (r) and P. (DOCX) [file pone.0096769.s008.docx]

**Table S6. Summary of median and IQR values for pooled data of CF and control subjects, with statistical comparison including values for the Mann-Whitney U (*U*), Z-score (*Z*), effect size (*r*) and *P*.**

| Variable | Pooled control | Pooled CF | *U* | *Z* | *r* | *P* |
| --- | --- | --- | --- | --- | --- | --- |
| F_0_ | 171.4  IQR 112.1 - 205.5 | 183.6  IQR 136.9 - 206.7 | 386 | -1.196 | -0.150 | 0.2343 |
| Intensity | 83.45  IQR 80.47 - 86.81 | 73.55  IQR 70.43 - 76.42 | 102 | -5.170 | -0.646 | < 0.0001* |
| Jitter | 0.3500  IQR 0.2900 – 0.4300 | 0.8100  IQR 0.4300 – 1.6300 | 100.5 | -5.193 | -0.649 | < 0.0001* |
| Shimmer | 0.3500  IQR 0.2450- 0.7750 | 1.3400  IQR 1.0400- 1.5100 | 123 | -4.877 | -0.610 | < 0.0001* |
| HNR | 13.650  IQR 10.610 – 18.200 | 6.280  IQR 4.190 – 10.470 | 191 | -3.925 | -0.491 | < 0.0001* |
| G | 1.0  IQR 0.0 - 1.0 | 1.0  IQR 1.0 - 3.0 | 253 | -3.242 | -0.405 | 0.0012* |
| R | 0.0  IQR 0.0 – 1.0 | 1.0  IQR 1.0 - 3.0 | 191 | -4.238 | -0.530 | < 0.0001* |
| B | 0.0  IQR 0.0 – 0.0 | 1.0  IQR 1.0 - 2.0 | 156.5 | -4.941 | -0.618 | < 0.0001* |
| A | 0.0  IQR 0.0 – 0.0 | 1.0  IQR 1.0 – 2.0 | 119.5 | -5.589 | -0.699 | < 0.0001* |
| S | 0.0  IQR 0.0 – 1.0 | 0.0  IQR 0.0 – 1.0 | 840.5 | 0.000 | 0.000 | 0.6393 |

*Statistically different according to the P<0.01 significance level and tested with the Mann-Whitney test.
