# Supplementary material for: Voice Disorder in Cystic Fibrosis Patients
Source: PLoS One. 2014 May 5;9(5):e96769. doi: 10.1371/journal.pone.0096769 (PMC4010511; doi:10.1371/journal.pone.0096769)
Supplement: Table S7 — Summary of median and IQR values for data of CF and control subjects segregated according to sex, with statistical comparison including values for the Mann-Whitney U (U), Z-score (Z), effect size (r) and P. (DOCX) [file pone.0096769.s009.docx]

**Table S7. Summary of median and IQR values for data of CF and control subjects segregated according to sex, with statistical comparison including values for the Mann-Whitney U (*U*), Z-score (*Z*), effect size (*r*) and *P*.**

| Variable | Female control | Female CF | *U* | *Z* | *r* | *P* | Male control | Male CF | *U* | *Z* | *r* | *P* |
| --- | --- | --- | --- | --- | --- | --- | --- | --- | --- | --- | --- | --- |
| F_0_ | 197.4  IQR 173.6 - 219.9 | 207.9  IQR 164.5 - 251.1 | 49 | -0.919 | -0.188 | 0.3746 | 125.2  IQR 102.5 - 174.9 | 164.0  IQR 128.1 – 186.8 | 127 | -1.690 | -0.267 | 0.0937 |
| Intensity | 82.68  IQR 80.47 - 86.65 | 75.93  IQR 73.41 - 81.18 | 17 | -2.878 | -0.588 | 0.0044* | 84.70  IQR 80.35 – 87.49 | 72.46  IQR 68.07 – 75.60 | 34 | -4.288 | -0.678 | < 0.0001* |
| Jitter | 0.3700  IQR 0.3025 – 0.4725 | 0.8850  IQR 0.5750 – 1.425 | 5 | -3.613 | -0.738 | 0.001* | 0.3400  IQR 0.2900 – 0.3950 | 0.7700  IQR 0.4100 – 1.710 | 46.5 | -3.941 | -0.623 | < 0.0001* |
| Shimmer | 0.2650  IQR 0.1750- 0.5100 | 1.440  IQR 1.235 – 1.528 | 1.5 | -3.829 | -0.782 | 0.0001* | 0.3600  IQR 0.3000 – 0.9750 | 1.290  IQR 0.4900 – 1.5100 | 83.5 | -2.906 | -0.460 | 0.0038* |
| HNR | 18.490  IQR 16.890 – 21.380 | 5.460  IQR 3.265 – 8.283 | 2 | -3.797 | -0.775 | 0.0002* | 11.78  IQR 9.950 – 14.270 | 7.070  IQR 4.190 – 18.880 | 124 | -1.774 | -0.280 | 0.0784 |
| G | 0.5  IQR 0.0 – 1.0 | 1.0  IQR 1.0 - 2.0 | 24 | -2.681 | -0.547 | 0.0081* | 1.0  IQR 0.0 – 1.0 | 2.0  IQR 1.0 - 3.0 | 112.5 | -2.194 | -0.347 | 0.0293 |
| R | 0.0  IQR 0.0 – 0.75 | 1.0  IQR 1.0 – 2.0 | 18 | -3.143 | -0.641 | 0.0019* | 1.0  IQR 0.0 – 1.0 | 2.0  IQR 1.0 – 3.0 | 85.5 | -3.034 | -0.480 | 0.0025* |
| B | 0.0  IQR 0.0 – 1.0 | 1.0  IQR 1.0 – 2.0 | 13 | -3.632 | -0.741 | 0.0003* | 0.0  IQR 0.0 – 0.5 | 2.0  IQR 1.0 – 3.0 | 73 | -3.525 | -0.557 | 0.0004* |
| A | 0.0  IQR 0.0 – 0.0 | 1.0  IQR 1.0 – 1.75 | 14.5 | -3.639 | -0.743 | 0.0003* | 0.0  IQR 0.0 – 0.0 | 1.0  IQR 1.0 – 3.0 | 202 | -4.895 | -0.502 | < 0.0001* |
| S | 0.0  IQR 0.0 – 1.0 | 1.0  IQR 0.0 – 1.0 | 41.5 | -1.591 | -0.325 | 0.1198 | 0.0  IQR 0.0 – 1.0 | 0.0  IQR 0.0 – 1.0 | 172 | -0.488 | -0.077 | 0.6368 |

*Statistically different according to the P<0.01 significance level and tested with the Mann-Whitney test.
